# Assessing clinical reasoning in the OSCE: pilot-testing a novel oral debrief exercise

**DOI:** 10.1186/s12909-023-04668-5

**Published:** 2023-10-03

**Authors:** Alexis Régent, Harish Thampy, Mini Singh

**Affiliations:** 1 Service de médecine interne, Centre de référence maladies auto-immunes et systémiques rares d’ile de France, APHP-CUP, Hôpital Cochin, F-75014 Paris, France; 2 Université de Paris, 15 rue de l’école de médecine, F-75006 Paris, France; 3 Division of Medical Education, Faculty of Medicine, Biology and Health, University of Manchester, Manchester, UK

**Keywords:** Clinical reasoning assessment, Oral debrief, Objective structured clinical examination

## Abstract

**Introduction:**

Clinical reasoning (CR) is a complex skill enabling transition from clinical novice to expert decision maker. The Objective Structured Clinical Examination (OSCE) is widely used to evaluate clinical competency, though there is limited literature exploring how this assessment is best used to assess CR skills. This proof-of-concept study explored the creation and pilot testing of a post-station CR assessment, named Oral Debrief (OD), in the context of undergraduate medical education.

**Methods:**

A modified-Delphi technique was used to create a standardised domain-based OD marking rubric encapsulating the key skills of CR that drew upon existing literature and our existing placement-based CR tool. 16 OSCE examiners were recruited to score three simulated OD recordings that were scripted to portray differing levels of competency. Adopting a think-aloud approach, examiners vocalised their thought processes while utilising the rubric to assess each video. Thereafter, semi-structured interviews explored examiners’ views on the OD approach. Recordings were transcribed, anonymised and analysed deductively and inductively for recurring themes. Additionally, inter-rater agreement of examiners’ scoring was determined using the Fleiss Kappa statistic both within group and in comparison to a reference examiner group.

**Results:**

The rubric achieved fair to good levels of inter-rater reliability metrics across its constituent domains and overall global judgement scales. Think-aloud scoring revealed that participating examiners considered several factors when scoring students’ CR abilities. This included the adoption of a confident structured approach, discriminating between relevant and less-relevant information, and the ability to prioritise and justify decision making. Furthermore, students’ CR skills were judged in light of potential risks to patient safety and examiners’ own illness scripts. Feedback from examiners indicated that whilst additional training in rubric usage would be beneficial, OD offered a positive approach for examining CR ability.

**Conclusion:**

This pilot study has demonstrated promising results for the use of a novel post-station OD task to evaluate medical students’ CR ability in the OSCE setting. Further work is now planned to evaluate how the OD approach can most effectively be implemented into routine assessment practice.

**Supplementary Information:**

The online version contains supplementary material available at 10.1186/s12909-023-04668-5.

## Introduction

Clinical reasoning (CR) is a complex skill defined as the cognitive operations that enable physicians to observe, collect, and analyse patient information resulting in clinical decisions that take into account a patient’s specific circumstances and preferences [1]. How clinicians think and reason can be described using the dual processing model [2]. System 1 thinking draws upon pattern recognition, experience and intuition to make quick, automatic and effortless clinical decisions. System 2 thinking on the other hand is slower and analytical, supporting hypothetico-deductive reasoning. The best predictor for successful (diagnostic) clinical reasoning is the quality of System 1 processing, in particular the probability of the correct diagnosis being considered by the clinician [3]. Whilst senior clinicians unconsciously toggle between both systems of thinking, junior trainees and students do not have enough clinical experience to solely rely upon system 1 thinking and should therefore be encouraged to also develop their system 2 problem-solving abilities [4, 5].

In supporting clinical learners transition from novices to expert decision makers, the early implementation of CR in undergraduate medical curriculum is recommended [6]. Indeed, the literature is rich with papers exploring curricular implementation of CR with myriad teaching interventions, ranging from stand-alone sessions through to longitudinal implementation, being described and evaluated [7, 8]. However, to achieve constructive alignment, it is also necessary to ensure that these skills are appropriately assessed.

Miller’s pyramid offers a useful framework from which to consider the range of possible assessment tools that target CR competency at different levels [9]. Assessments at the lowest two levels (“knows” and “knows how”) evaluate underpinning knowledge and knowledge application. In the context of CR, potential examples of assessment tools that function at this level include key feature problems and script concordance tests [10, 11]. Assessments at the “shows how” level of the pyramid evaluate learners’ ability to demonstrate competency across a range of skills. The Objective Structured Clinical Examination (OSCE) is typically seen as the gold-standard approach at this level [12], though as will be described later, has arguably not been used to its full advantage to assess CR skills [13].

Lastly, at the ‘does’ level, learners are evaluated using workplace-based assessment tools to determine whether they can continue to demonstrate competency in the in-vivo setting of clinical workplace environments. CR-focussed tools such as SNAPPS and the Assessment of Reasoning Tool have been described at this level [14, 15].

Despite its ubiquitous application across the spectrum of medical education, little attention has been paid to how OSCEs can be best designed to assess students’ CR. OSCEs require candidates to participate in a series of simulated scenarios that are each assessed by trained examiners using standardised scoring rubrics. In the authors’ experience, OSCE stations are typically scripted to provide stereotypical representations of patient conditions leading candidates to a single, most likely diagnosis, but in doing so, strongly favour system 1 pattern recognition approaches. Instead, it has been suggested that stations should be designed to better reflect authentic clinical presentations in which a range of plausible differentials are generated [13]. Furthermore, it is recognised that OSCEs tend to focus more on the ‘what’ of decision making (generating potential diagnoses or management plans) over the ‘how and why’ (the ability to explain one’s analytic approach to then justify and prioritise diagnostic and management decisions i.e. demonstrating CR processes). It is assumed that the OSCE candidate who is taking a structured history and arrives at a reasonable differential diagnosis is applying sound reasoning processes. However, this assumption is easily challenged. Candidates can apply consultation frameworks to collect cues and present data without employing analytical data processes – in effect simply ‘interviewing’ the patient using set of questions devoid of a purposeful reasoning approach. Furthermore, a candidate who offers a reasonable differential diagnosis at the end of the station may do so without relating the data they gained during the consultation, either through chance or simple guesswork based on epidemiologically likely diagnoses ascertained from the presenting symptom(s). These concerns are supported in studies demonstrating weak or absent correlations between how students are scored whilst being observed taking a history to how they are judged when asked to subsequently list, prioritise and justify possible differential diagnoses arising from that consultation in a written post-station encounter exercise [16]. Therefore, OSCE candidates’ application of CR is typically not assessed, with predominance given to observation of what the learner does and says rather than reasoning approach they use.

Recognising these limitations, recent work has explored ways to more effectively evaluate a candidate’s CR processes within the OSCE format. Much of this work focuses on the use of post-encounter forms in which candidates complete a brief written exercise following a consultation station where they describe and justify their diagnostic reasoning. Whilst post-encounter forms offer advancement in CR evaluation within the OSCE context, they are limited by the use of pre-set questions and the written format restricting opportunities to further probe candidates’ responses.

This pilot proof-of-concept study therefore set out to explore an alternative post-encounter CR OSCE task, named Oral Debrief (OD) for final year medical students at a large UK medical school. It builds upon previous work describing the implementation of a longitudinal CR curriculum within our large undergraduate medical programme [8] in which our students’ reasoning skills are regularly evaluated within rotating clerkships using the Manchester CR tool (MCRT).

The concept of OD to explore a student’s CR skills in the OSCE arose from the written post-encounter forms previously described [16] and the recognised importance of preceptor-student discussions within workplace-based CR assessment [17, 18]. It is proposed as a post-history station verbal exercise in which the examiner uses semi-structured question prompts to explore a candidate’s clinical reasoning ability and scores them using a standardised marking rubric.

In our main research question, we set out to answer whether a post-encounter OD could be used identify differing levels of medical student CR ability.

To answer this core question, we first created a standardised marking rubric encapsulating the key skills of CR that could be used to score ODs. Using pre-scripted video-recorded OD performances, secondary questions explored:

1. What do examiners look for during an OD when marking candidates’ CR ability using this rubric ?
2. What are examiners’ views of assessing CR skills using the OD approach ?
3. Can the marking of a candidates’ OD performance can be independently conducted from the marking of the preceding linked history-taking station ?

## Methods

### Development of the OD approach and marking rubric

**Fig. 1:**
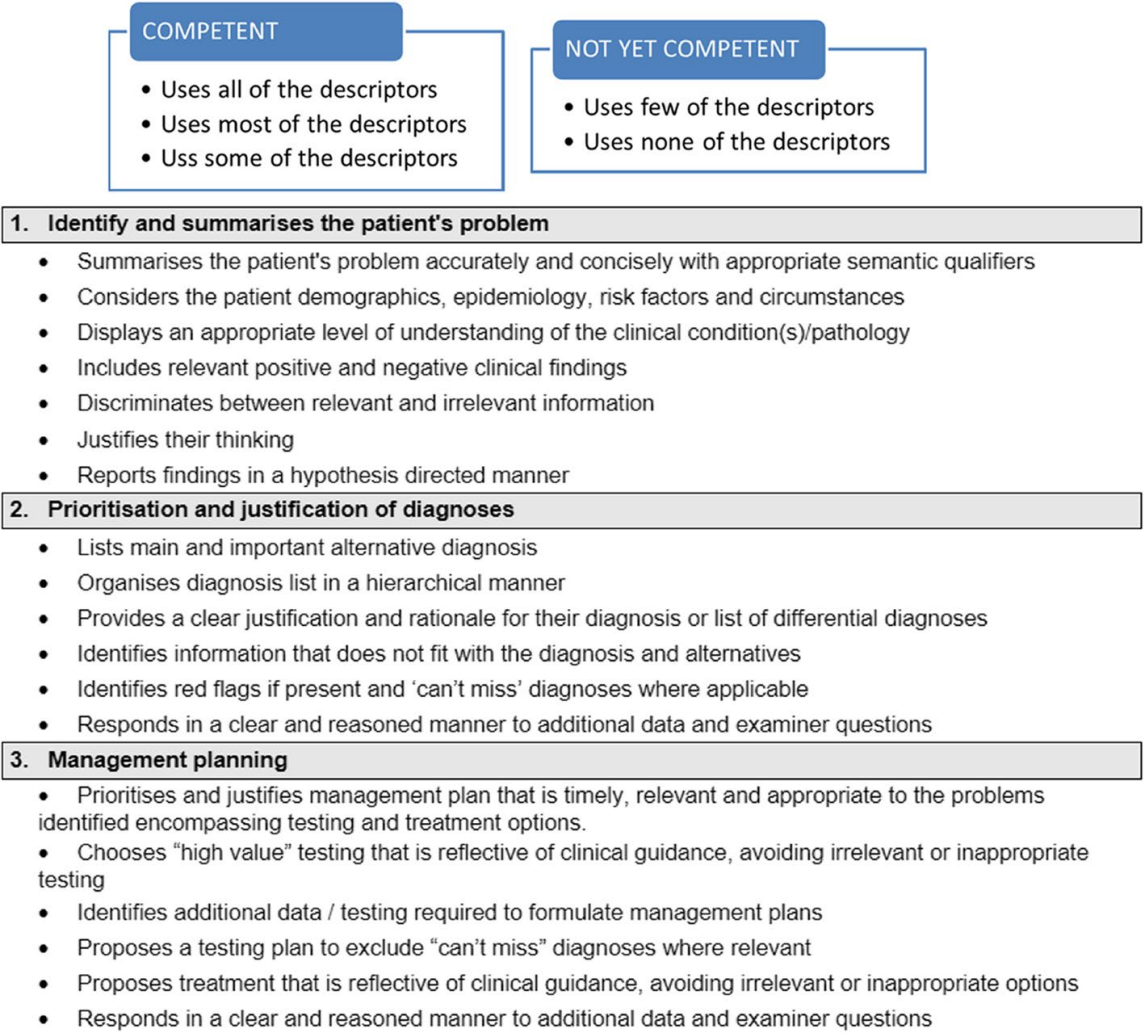
Overall global judgment scale

The OD was designed as an 8-min post history-taking station task. In line with the existing OSCE marking method a domain-based rubric with an overall global judgement scale was designed to maintaining candidates’ familiarity with the assessment approach. Using the MCRT as a starting point, a Delphi-based approach [19] involving all members of the school’s established CR working group was conducted to agree domains and associated descriptors to reflect the key skills of CR. The group consists of seven clinical academics with interests in CR pedagogy and who collectively implemented, and now maintain, the aforementioned longitudinal programmatic integration of CR within the undergraduate medical curriculum. In addition to domain marking, the OD rubric utilised an overall global judgement score adopted unchanged from our programme’s existing OSCE assessment scale as shown in Fig. 1.

### Development of scripted OD performances

To evaluate how well this marking rubric worked in differentiating students’ CR abilities, a series of videos were created demonstrating an OD task in action. To achieve this, we first developed a history-taking station for a patient presenting with abdominal pain and change in bowel habit. The patient history was carefully crafted to generate several plausible differentials and thus require analytical system 2 based thinking (for the station script please see Supplemental file 1). Student and examiner volunteers were recruited to produce simulated recordings of the OD which were conducted on Zoom™ given ongoing exigencies of the covid pandemic. Three differing global judgement levels of OD performances were scripted to portray the failing, satisfactory and excellent candidate. Whilst each script utilised the same core set of examiner prompts for standardisation, the simulated examiner was permitted to clarify and explore specific areas as felt required(provided in Supplemental file 2). Following initial run-throughs to achieve appropriate levels of performance, the set of ODs were then recorded, reviewed and edited by the authors. The history-taking station task itself was then recorded to depict a satisfactory candidate to be shown to participants after they scored the OD tasks as detailed later.

### Testing the marking rubric

Having developed the OD marking rubric and the example OD videos, clinicians from our existing bank of OSCE examiners were invited by email to a one-hour interview conducted on Zoom™ in which they watched three recorded ODs and scored these against the rubric followed by participation in a semi-structured individual interview. Using a convenience sampling approach, all sixteen examiners who agreed to participate were recruited. Participation was entirely voluntary.

Prior to attending the interview, participants were emailed the history-taking station script that formed the basis of the OD scenarios to review in advance. To avoid participants assuming that their selected three videos would portray each of the three levels of performance and thus influence how they marked, we had recorded two videos for each of the three levels of performance. A randomisation matrix was used to provide a set of three videos to each participant. Some participants were allocated one video from each level whilst other were provided two videos of one level and one of another. The allocation approach was not disclosed to participants.

The ‘think-aloud’ method [20] was used as participants watched each OD, enabling them to verbalise their thoughts as they watched each video, providing insight into their thought processes, perspectives and judgement-making approach as they assessed students’ CR ability. Participants were able to pause each video to share their observations and explain their scoring.

Following the think aloud stage, participants were then asked to score each candidate’s performance across the rubric domains and overall global judgement. To evaluate if scoring of OD could be conducted independently to that of the preceding history-taking station, participants were shown this recording only after scoring the ODs. Participants were asked if this would change their initial OD scores.

Lastly, participants took part in a semi-structured interview (topic guide provided in Supplemental file 3) to explore their experiences more generally in using the OD marking rubric and to reflect upon the inter-dependency of the OD scoring from the history-taking task itself.

Alongside the participant tasks above, all seven members of the Manchester CR Group (hereafter named ‘CR examiner group’). were asked to independently score each video to a) confirm that the OD recordings did indeed portray their intended level of performance and b) to further determine inter-rater reliability metrics of the rubric.

### Ethical considerations

Completion of our institution’s research ethics decision tool confirmed that this work was exempt from formal review as it constituted programme evaluation with no involvement of sensitive/confidential information nor use of vulnerable groups. We did however throughout this work pay attention to ethical considerations and followed the principles of the Declaration of Helsinki on research participants. Participation was entirely voluntary with no repercussions for non-participation. The audio was submitted for transcription with all personal identifiable information removed. All participants were provided with a detailed participant information sheet and asked to sign a Informed consent form before taking part including informed consent for participation and publication of anonymised quotes.

### Analysis

#### Analysis of scoring

**Fig. 2:**
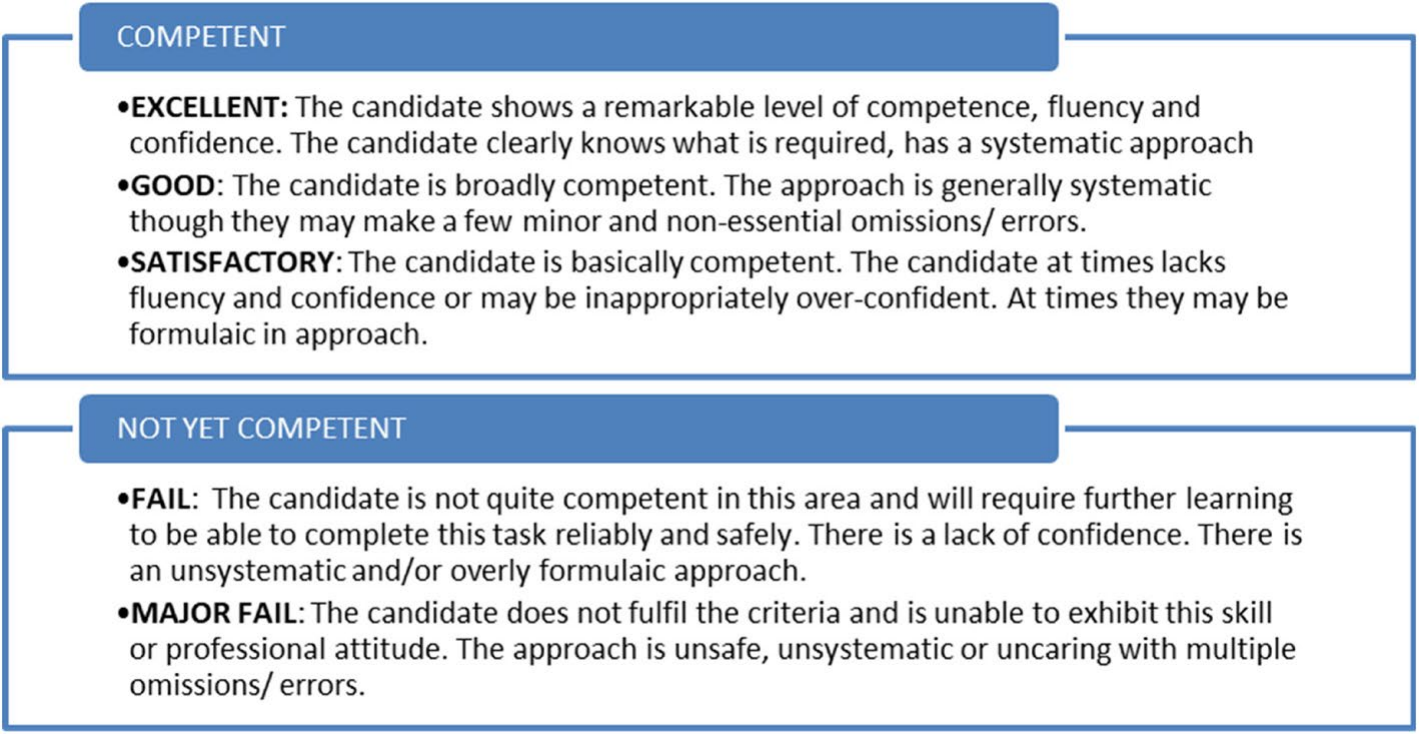
OD Domains and corresponding descriptors. For each domain, examiners scored the candidate’s performance against the following scale

As part of determining the utility of the marking rubric, the Fleiss’ kappa statistic, indicating inter-rater reliability, was calculated using MS Excel. This was conducted both within the sixteen participant ‘typical examiner’ group and within the seven ‘CR examiner’ group. Given the small number of scorers involved, analysis focussed on the broader binary categorisation that examiners initially undertake in differentiating between competent and not-yet competent candidates for both domain scales and overall global judgement (Figs. 1 and 2). In line with published interpretation guides, a Fleiss Kappa of 0.21–0.4 was denoted to indicate fair agreement, 0.41–0.6 moderate agreement, 0.61–0.8 good agreement, and above 0.8 very good agreement [21].

#### Thematic analysis

Each interview was audio-recorded and anonymised prior to transcription. A reflexive thematic analytical approach was taken following the steps of Braun and Clarke [22]. Transcripts were initially coded both inductively and deductively to highlight key aspects of participants thinking as they scored, including recurring or contrary views. Thereafter all codes were reviewed in an iterative fashion to generate overarching explanatory themes.

## Results

### Delphi-based production of marking rubric

Through iterative rounds conducted over three months, consensus agreement was achieved in creating a set of three CR skill domains namely, “Identification and summarisation of the patient’s problem”, “Prioritisation and justification of diagnoses” and “Management planning”. For each domain, agreement was also reached for component descriptors for each domain detailing expected elements to be demonstrated. The domains and descriptors are listed in Fig. 2.

### Analysis of scoring

The seven-member ‘CR examiner’ group generated 42 assessments, each scoring all 6 OD videos whilst the sixteen-member participant examiner group generated 48 assessments, each scoring 3 OD videos.

**Fig. 3:**
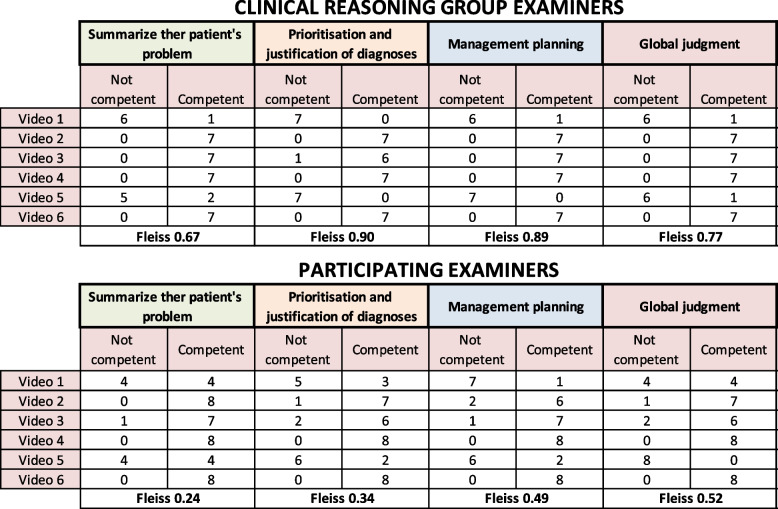
Analysis of OD scoring using the Fleiss kappa inter-rater reliability statistic

The CR examiner group demonstrated strong inter-rater reliability with good and very good Fleiss kappa statistics seen across domains and global judgement. Their scoring aligned to the intended level of portrayal for each video, confirming that these had been appropriately scripted. The participant examiner group showed lower Kappa values though still achieved fair to good agreement for the three domains with good agreement for the global judgement. Figure 3 details the breakdown of agreement by group and by video.

### Thematic analysis of think-aloud scoring

Thematic analysis of think-aloud transcripts generated three themes that described the decision-making process participants undertook when deliberating upon a candidate’s display of clinical reasoning during the OD task. The first theme describes cognitive aspects of the candidate that were considered during scoring. The second theme focuses on how candidates translated these cognitive aspects into a verbalised demonstration of their CR skills. The third theme reflects the influences that participants’ own reasoning and clinical/assessor background had on their scoring of candidates. Each theme was enriched using component sub-themes generated from the coding structure and is summarised in Table 1 with exemplar quotes provided.

**Table 1:** Themes, subthemes and corresponding exemplar data

Theme	Subtheme	Exemplar quotes
Cognitive aspects relating to the candidate	Knowledge	“she appears to be covering for a lack of knowledge” “He shows a very deep knowledge of the GI system which I’m impressed with. He seems very sensible, seems very knowledgeable” “She literally only said about three things.. there’s many more things than that” “he’s not really taken a proper history of the characteristics of the pain or the presentation”
	Reporting information obtained from the preceding station	“Although I’ve not seen the history-taking itself, I can see that it’s obvious that she has explored other systems” “She wasn’t able to demonstrate to me that she had really taken all of the history into account during the history taking process” “He was excellent and he told me in his differential that he did a systems review…but I didn’t observe him doing that, so I don’t really know what he asked, or whether he did ask the relevant questions”
	Analysis & discrimination	“She’s just sort of taking everything at face value and not picking and pushing a bit more” “Its very superficial, very superficial account but then she’s been inappropriately specific and making assumptions” “She’s only concentrating on one diagnosis.. she didn’t give me enough negatives or positives in her history taking”
How candidates demonstrate their CR through what they do in the OD	Adopting structured approach	”This student has started with a very clear, concise and accurate summary of the history” “she’s kind of jumping around a bit there. She started off being quite structured in her answer and now she’s realised stuff that she hasn’t mentioned in her summary “[he has an] unsystematic approach… it sounds good, but in fact when you listen to it carefully it’s- he bounces from idea to idea…an awful lot of words to say but very limited information”
	Ability to prioritise & justify	“[The student] organised these [differential diagnoses] at the very outset in a hierarchical manner from most likely, serious, and down to the least likely with a very good justification each time” “I like the way she using kind of explanations as to why she thinks things are a top differential” “She doesn’t seem to me to be really thinking about what her differentials are and how she can start to prioritise them and think in an ordered way around what her differentials would be.” “Not really telling me how it would, how she would differentiate between any of her differentials”
	Attention to patient safety	“They’ve dismissed the weight loss, which is never a good thing to do” “Is he going to be a safe pair of hands? This is what you think of with a global judgement” "it’s good to talk about safety-netting… and he’s specifically now mentioned red flags”
	Who led the OD	“There's a lot of prompting around trying to extract this” “He's not generating anything really without prompts, and even with prompting, he's giving very short and answers, not going into again”
	Candidates’ confidence & presentation style	“He’s being very clear about this thought processes, which is really useful as an examiner to know, in terms of what your approach was” “He’s got a nice, precise presentation style” “The way that she’s presenting the history seems like I can sort of see what she’s thinking behind why she’s saying what she’s saying” “He seems a bit flustered and not answering well… it’s not showing very ordered thinking “It was obvious to me that she just popped it in there because she was just kind of going like randomly going through what she said “I feel like my kid would be able to give a better like summary than this student”
	Examiners’ questioning stye and approach / idiosyncrasies	“I don’t know whether [the candidate] thought that at the time and discounted it…or if she’s just thinking of it now that [the examiner] said it, and working through the thought process live now” “That was a leading question from the examiner which should, she should have brought it out herself… So I’m a little disappointed that there was a leading question there” “I thought the examiner was a much more helpful examiner as well as helping her lead, giving her some leading questions of stuff that she’d missed off”
Cognitive aspects relating to the participant examiner	Examiners own clinical background as frame-of-reference	“I wouldn’t have musculoskeletal pain at all in my differentials” “If I’d been examining the scenario, I don’t think I’d have picked up that connection for her considering it with the infection” “So what I expect of myself is actually what I would expect of a medical student” “I may personally be quite fixated on endometriosis because of the association with the patient’s period. But then again I’m not a specialist in endometriosis and I’m not sure how it presents symptomatically”

#### Cognitive aspects relating to the candidate

Participants expressed that a fundamental element that underpinned candidates’ reasoning approach was their level of background knowledge and areas of potential knowledge deficit. Comments about candidates’ knowledge ranged from their understanding of pathology, epidemiology and symptomatology for particular conditions through to how this knowledge was then not only extended to, but also compared and contrasted with, understanding of associated conditions. Having a sufficiently robust baseline level of knowledge was seen to be a core pre-requisite upon which to apply reasoning skills. The second cognitive aspect related to how candidates internally analysed and then reported the data they obtained through the preceding history station. Participants in this study were asked to observe the OD without having seen how the candidate performed in the history task itself. As such, participant comments revealed how they speculated about the veracity and accuracy of comments made by candidates about what they had asked during the history and what information this elicited. Inferences were made as to how well candidates had utilised their reasoning skills in the history-taking station itself in terms of whether they had followed a purposeful questioning approach to narrow down the differential diagnosis.

#### How candidates demonstrate their CR through what they do in the OD

Participants referred to what they saw the candidate do or say during the OD as a window into their clinical reasoning ability. These factors could be categorised into those that directly influenced the scoring and those that influenced how CR skills were being shown through the OD task itself.

In terms of the former, participants opined that candidates who were seen to adopt a structured approach in presenting the case with the use of appropriate semantic qualifiers were displaying higher levels of CR ability than those who did not. In particular, participants perceived CR was more strongly displayed when candidates successfully discriminated between relevant and less relevant information and in doing so, prioritised and justified their decisions. Aside from what was being said by the candidate, participants also considered who led the OD discussions. Candidates who led the conversation and anticipated what was due to be asked in their responses, negated the need for additional questions to be asked by the examiner and were seen to display higher levels of CR ability.

Attendance to patient safety appeared to be a key marker of CR ability for participants such as considering ‘red flag’ diagnoses or potential harm from investigative or management options. Additional factors that influenced their judgements included how confident the candidate appeared and how articulate they were in verbalising their thinking process during the OD exercise. Generally, participants associated higher levels of CR ability with candidates who adopted a confident presentation approach. Other participants however recognised that this was more complex and a less-confident candidate may be reflecting the anxiety of the assessment environment rather than weaker CR skills.

The second category related to how the OD delivery approach influenced how candidates displayed their CR skills, which in turn potentially affected how they were scored. Particularly, participants felt that the questioning style adopted by the video examiners affected how candidates then responded with comments made about word choice, clarity and the tone. They reflected that this may have been attributable to examiner’s idiosyncrasies, recognising that different examiners had differing approaches to exploring the same part of the OD. Participants also suggested that the recorded examiners own clinical background and assessor experience may have influenced what questions were posed and how these were asked.

#### Cognitive aspects relating to the participant examiner

Participants frequently drew upon their own clinical expertise as a frame-of-reference in judging the students’ CR skills, often referring to their own ideas about potential differential diagnoses and management approaches. For examiners who had a clinical background aligned to the station topic, this enabled a deeper critique of candidates’ responses. Conversely, examiners who were less clinically familiar with the topic voiced discomfort and difficulty in determining if candidates’ verbalised reasoning was in line with current clinical practice. Furthermore, during the think-aloud, examiners referred to the history-taking station content itself, comparing candidates’ reports on how they elicited the history with how they believed they themselves would have performed. They again relied upon their own clinical knowledge of the area to guide this comparison.

### Participants’ view on OD

The majority of the participants (11/16) found the marking rubric useful and reported increasing confidence in applying the rubric as they watched more recordings. 14 examiners agreed that the OD exercise was a direct assessment of CR skills whilst 2 examiners felt it reflected more a student’s presentation ability. Some suggestions were made during interviews to improve the domain descriptors such as adding in a requirement to mention absence/presence of red flag symptoms and adding in timescales within the ‘management planning’ domain. Participants also recognised that each descriptor and each domain were not necessarily equally weighted in arriving at their global judgement but felt generally confident in making this overall decision.

Participants also considered how the OD might be delivered in routine OSCE assessments. They raised aforementioned concerns that the way in which examiners used the semi-structured prompts to conduct the exercise may have influenced candidates’ performance and therefore scoring. There was consensus that OD could serve as an effective summative tool for assessment of medical students’ CR ability if implementation included a prior transitional formative phase to enable both students and examiners to become familiar with the methodology.

### Inter-dependency of scoring OD and the prior station

**Fig. 4:**
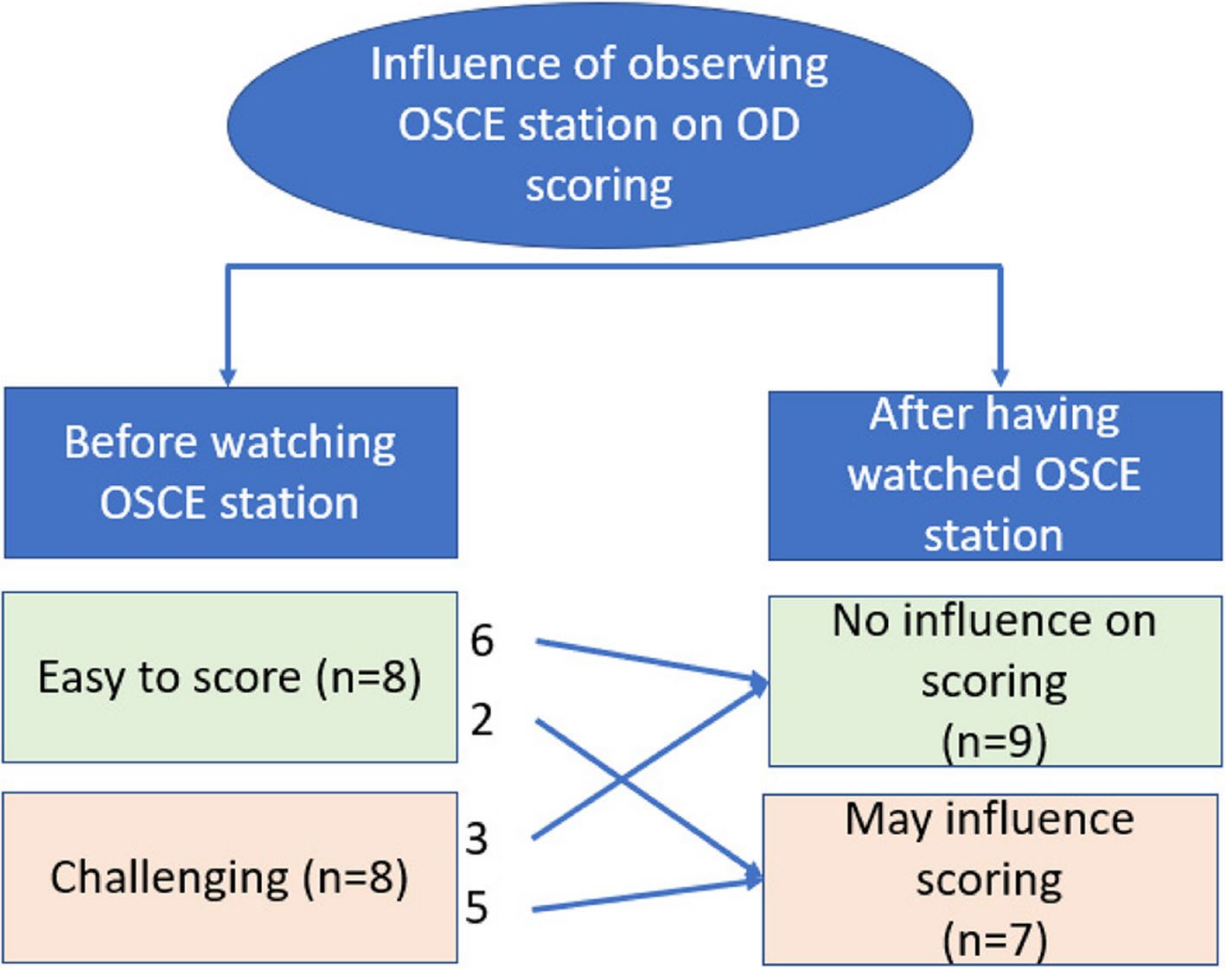
Exploration of the inter-dependency of how candidates perform in the OSCE station itself with how they were scored in the OD

To determine if the OD could be marked independently to the linked history-taking station, examiners scored candidates’ each OD without being shown how they had performed in the prior station. Half of the examiners reported this was not detrimental to their scoring suggesting independence of these two tasks. The other half however felt that this created additional challenges for them, generating speculation about what the student had claimed they had asked the patient and what the patient had said. Towards the end of the of interview, participants were shown a prior history-station recording that corresponded to one of their three ODs and were asked to reflect if this would change their scoring. As shown in Fig. 4, just over half of all participants did not feel a need to adjust their OD scoring having subsequently watched the initial OSCE station task itself, though the majority of these were participants who initially found the OD easy to score. Conversely, those who viewed the OD more challenging to score were more likely to feel that watching the OSCE station would change the scores they had awarded for the OD component.

## Discussion

**Fig. 5:**
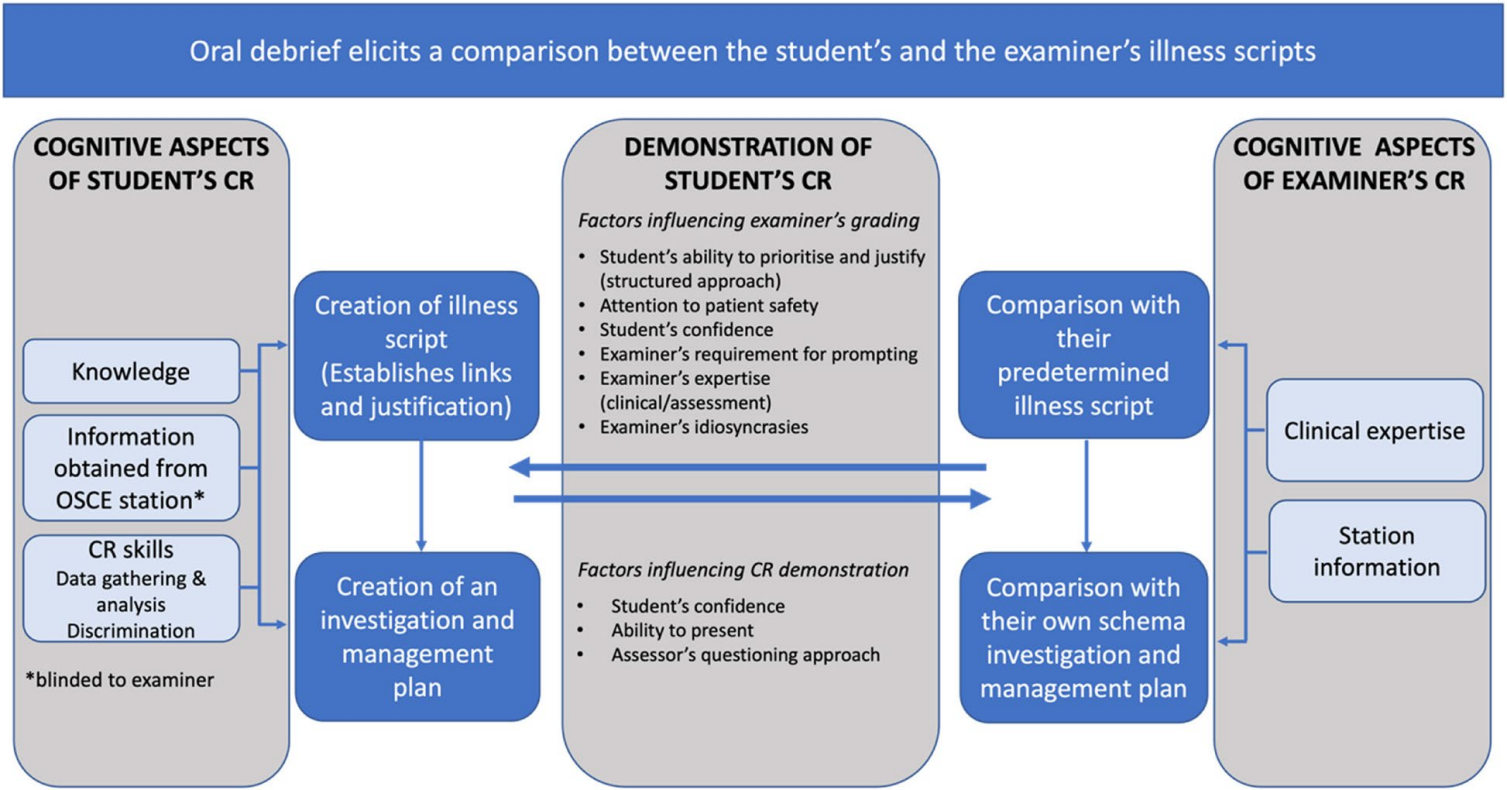
Proposed cognitive model describing how CR ability is scored using the OD approach

The findings of this proof-of-concept study provide initial evidence for the role of a post-history taking CR exercise named oral debrief that explores candidates’ verbal report of their reasoning approach and clinical decision making. Through the think-aloud interviews, several factors were identified that participating examiners considered when scoring candidates abilities. Figure 5 displays these in a proposed cognitive model [23] mapped to the three generated themes. Both candidates’ and the participating examiners’ own cognitive processes were underpinning foundations from which the oral debrief was conducted in which candidates were required to translate their thinking into verbalised responses whilst examiners steered the discussions through questioning based on their own reasoning approach. In doing so, the OD elicited comparisons between the candidate’s and examiner’s illness scripts, a term that describes how clinical knowledge is stored, organised and retrieved [24]. The identified factors spanned those that indicated higher CR abilities and markers of a poor reasoning approach. Within these factors, participants paid particular attention to patient safety implications within candidates’ responses. The ability to discriminate between important and less important data, present in a structured fashion using sematic qualifiers, and the ability to prioritise and justify decisions were other key CR skills that informed scoring.

The ‘utility equation’ proposed by van der Vleuten [25] offers a useful framework upon which to critically expand further upon these findings. The utility equation suggests there are five core components of any assessment that should be considered for the overall approach to be considered useful, namely: validity, reliability, educational impact, feasibility/ cost and acceptability.

A valid assessment achieves what it is intended to measure and can be explored through its differing facets. Content validity is the degree to which an assessment represents all aspects of tasks within the area being assessed. This was achieved through the Delphi-based approach in which three overarching domains encapsulating the core stages of CR were designed. To attain construct validity, our study methods ensured CR was appropriately represented through detailed descriptors reflecting the key expected CR skills for each domain. Construct validity also links to the concept of constructive alignment [26] in which assessment is linked to teaching & learning activities and intended learning outcomes. Our undergraduate programme places particular emphasis on developing clinical reasoning skills using regular classroom and experiential-based learning activities supplemented by frequent formative assessment within clinical placement rotations using the Manchester Clinical Reasoning Tool [8]. In designing the OD rubric, the MCRT formed the basis upon which the final rubric was developed and thus maintained candidates’ familiarity with core expectations. Lastly, face validity was judged by seeking the views of the participant examiners, the majority of whom agreed that the OD approach offered an effective way to assess a candidate’s CR abilities. It is acknowledged that further work is now needed to establish wider stakeholder views, particularly from candidates.

Reliability reflects the degree to which the scoring instrument can reproduce results. Despite the small-scale pilot nature of this work, the rubric demonstrated reasonable inter-rater reliability metrics for both participant and CR examiner groups suggesting that the domains and global judgement were appropriately designed to adequately discriminate between competent and not-yet competent candidates. It was clear however that the ‘expert group’ demonstrated higher levels of agreement across the rubric scales for all videos. There are several potential explanations for this. The expert group were also those who conducted the Delphi exercise and thus had intimate knowledge of the scoring rubric and detailed understanding of the OD approach and aims. Participating ‘typical’ examiners on the other hand were asked to use the rubric without any specific training aside from being informed it was to be used to score aspects of observed CR. Secondly, the two videos that particularly reduced the inter-rater reliability Kappa statistic for the participating examiner group were the two ODs scripted to depict the failing candidate. There is well-recognised ‘failure to fail’ phenomenon in assessment [27] which may have resulted in more divergent scoring for this group. These findings therefore highlight the importance of delivering OD specific training to develop assessor expertise and more consistent scoring. A crucial part of maintaining reliability is standardisation of the assessment approach. In scripting the videos, the simulated examiners were provided with a pre-defined list of question prompts that they could use to facilitate the OD exercise. This ability for examiners to probe and explore candidates’ thinking, during OD, was a key development designed to overcome the restricted format of existing written post-encounter forms. However, the findings highlight that the way in which examiners asked these questions may have influenced how candidates then performed. This is a concern in future implementation, especially if examiners decreasingly rely on these prompts in exchange for adopting their own questioning approach. Further work is now planned to pilot-test the OD approach in a formative OSCE to evaluate the degree to which examiners use these question prompts and how reliability can be maintained whilst retaining the key benefit of OD which is deeper exploration of a candidate’s thinking through flexible questioning.

Feasibility refers to the practical logistical considerations to an assessment approach. We delivered the OD as a resource-light 8-min post-station task allowing this to easily be substituted for a traditional station without the need to significantly alter existing delivery approaches. It is acknowledged however that doing so without an increase in total number of stations may impact on the sampling blueprint of the overall exam. Furthermore, reasoning skills are considered context-specific i.e. the ability to demonstrate reasoning in one clinical scenario does not necessarily mean the candidate has the ability to do so in another [28]. Programmes should therefore consider OD implementation across clinical station scenarios. It remains unclear if a different examiner to the individual who assessed the preceding linked history-taking station can conduct OD as noted in this study. Further work is needed in this regard to determine the inter-dependency of these two stages using multiple candidates and stations. The result of this will determine if the station could be run as a double-length station with the same examiner or as a separate task with a different examiner as was modelled in this pilot.

For an assessment to have educational impact, it should encourage deep-learning; assessment not only *of* learning, but assessment *for* learning [29]. The OD approach is modelled on the workplace-based formative CR tool already in use at our institution and so was deliberately designed to stimulate students’ engagement with CR classroom activities and experiential learning encounters. By expanding the OSCE focus beyond the what, to the why and how, the OD approach is likely to further encourage learners to develop their reasoning abilities throughout their clinical placements. Educational impact of the OD approach may also be enhanced by examiners providing narrative comments on areas of strength and future development in addition to domain and global judgement scores. To stimulate future practice, feedforward that provide specific constructive comments on what the learner should focus on next, and how to achieve this, will offer most impact [30].

The remaining component of the utility equation, acceptability, reflects the importance of ensuring that the assessment method is acceptable to all stakeholders. In this study, this was only evaluated from the participant examiner perspective with no concerns reported about the proposed approach either for the examiners themselves, nor any perceived possible adverse implications for candidates identified. Participants did report increasing comfort scoring using the rubric as they progressed through all three videos again highlighting the importance of providing sufficient examiner training on the OD methodology in order to clarify the expected aims of the task, how to maintain standardisation during the OD discussions and how to score consistently using the rubric [31].

## Limitations

Findings from this proof-of-concept pilot exploration will guide further larger-scale studies to further validate the OD approach. We recognise that our analysis did not explore other observed aspects of the candidate beyond CR ability, such as demographics, which may have influenced the awarded scores and thefore additional work is needed to explore this further. Inter-rater agreement was calculated at the broader level of differentiating competent from not-yet-competent students for domains and global judgement which was appropriate given the exploratory nature of this work. Future work with larger participant cohorts is needed to evaluate inter-rater agreement across the full range of the scale. Lastly, the focus of this OD approach was to explore candidates’ CR skills in the context of history-taking stations. There are however other OSCE station types and therefore exploration of how best to integrate CR assessments into these is warranted.

## Conclusions

This exploratory study offers emerging evidence to support the use of a post-station oral debrief exercise to assess medical students’ clinical reasoning skills in the OSCE setting. This evolves the OSCE from its current focus on assessing what is said and done, to exploring the why and how of a candidate’s performance. This would afford undergraduate medical programmes the crucial ability to explore underpinning analytical reasoning approaches used by candidates when making diagnostic or management decisions. The findings successfully provide a proof-of-concept upon which further work is now needed to investigate how this can most effectively be implemented into routine assessment practice.

### Supplementary Information

**Additional file 1.** OSCE station details

**Additional file 2.** Oral debrief scripted questions

**Additional file 3.** Individual interview semi-structured topic guide
