# Supplementary material for: Assessing clinical reasoning in the OSCE: pilot-testing a novel oral debrief exercise
Source: BMC Med Educ. 2023 Oct 3;23:718. doi: 10.1186/s12909-023-04668-5 (PMC10548592; doi:10.1186/s12909-023-04668-5)
Supplement: Supplementary file 1 — Additional file 1. OSCE station details [file 12909_2023_4668_MOESM1_ESM.docx]

**Supplemental File 1. OSCE station details**

**Instructions to Candidates**

You are a Foundation Doctor in General Practice.

Emily Watson, a 27-year-old female has presented with 6 weeks of abdominal pain.

Please take a history.

In the next station (Oral Debrief), the examiner will ask you for a summary of the patient’s problem, differential diagnosis and management plan.

**Information for the Examiner**

This is a station designed to test the candidate’s ability to take a history from a young woman with chronic abdominal pain and discuss differential diagnoses/ investigations.

The candidate should ask a few pertinent questions such as:

- Presenting Complaint: what is the symptoms(s), when did it start, how has it changed, site, onset, character, radiation, triggers, relieving factors, duration etc.
- Explore associated symptoms relevant to the presenting complaint: nausea, vomiting, bowels, fever, frequency of bowel movement, blood or mucus in stool, appetite, anal pain, mouth ulcers, rashes
- Exclude red flags: eg *weight loss, proctal bleeding*
- Gynaecological symptoms such as regularity of periods including last menstrual period, Intermenstrual bleeding, postcoital bleeding, dyspareunia
- Travel history
- Past medical history
- Drug history
- Alcohol (and smoking) status

**Information for Standardized Patient**

*Opening statement*

“I keep feeling pains in my tummy”.

*About You*

You are Emily Watson, aged 27 years.

You work in an office as an auditor.

You work at a computer most of the day.

You live with your husband and you do not report at risk sexual behaviour. You have no children.

You are non-smoker. You drink a glass or two of wine on a Friday and Saturday.

You have a fat diet and are thin: 55 kg 1m70

Your mood is normal (not anxious or depressed).

*The Situation*

You have suffered from abdominal pain in the last 6 weeks.

**If asked specifically:**

The pain is moderate 4/10. Pain varies during day/night.

Upper and lower part of tummy, most often middle part. Moving around can make it worse but you have not noticed any particular movements which is worse/better than others. The pain is there all the time – it feels like a dull ache but can be sharp at times: pain comes in waves of severe pain then goes on its own and comes back soon after. Nothing makes it better, and it was worse during the menstruation (you have had one period since the pain started). Opening your bowels only brings a modest relief.

You sometimes wake up during the nights (once every two or three nights). When the pain comes on, you take paracetamol though this only brings a modest pain relief for a few hours. For the past week you have been taking 2 tablets three times a day. Sometimes you have took ibuprofen in addition to paracetamol (you took this for around 3 days last week). You had first smear when you were 25 which was normal. Your periods are regular, every 4 weeks. Your last period 3 weeks ago was more painful than usual but lasted the same as it normally would, around 5 days. You don’t have particularly heavy bleeding and would normally use around 4 pads a day for the first 3 days and 2 a day for the next 2 days. Your last period though was heavier using around 5 pads a day for most of the period. You have not noticed passed any blood clots. You have not noticed any bleeding between periods or after sex.

You use condoms but no other contraception.

On around 2-3 occasions in the last 2 months, you have felt uncomfortable down below after sex – more internally than externally.

You have not been abroad recently apart from a week in Spain almost a year ago.

**You have not had:**

No painful, red or dry eyes.

Any skin conditions or rashes such as psoriasis and you have no family history of this.

No nail changes.

No urinary symptom.

No back pain, no other join pain.

No respiratory symptoms.

*Previous medical and surgical history:*

You had glandular fever when you were 18.

Otitis media 2-3 month ago, resolved completely.

Had once (4 years ago) an anal painful lesion while sitting.

*Medical problems in the family:*

Your mother and father are both alive and reasonably well.

Mother was diagnosed with hypothyroidism recently.

Your brother Anthony has type 1 diabetes and is on insulin.

*Medication*

None, paracetamol recently and ibuprofen over the counter in the last 6 weeks

Amoxicillin 2-3 month ago (Otitis media).

You do not take any alternative medicine.

You do not take recreational drugs.

You have no known drug allergies.

*How to play the role*

You are generally easy to talk to.

You give information freely if you feel you have been asked appropriately.
