# Supplementary material for: Assessing clinical reasoning in the OSCE: pilot-testing a novel oral debrief exercise
Source: BMC Med Educ. 2023 Oct 3;23:718. doi: 10.1186/s12909-023-04668-5 (PMC10548592; doi:10.1186/s12909-023-04668-5)
Supplement: Supplementary file 2 — Additional file 2. Oral debrief scripted questions [file 12909_2023_4668_MOESM2_ESM.docx]

**Supplemental File 2. Scripted questions to be used by the examiner during Oral Debrief**

1. Please summarise the patient history.
   - If not covered please ask the candidate to elaborate on: symptoms, demographics, risk factors.
2. Talk me through the approach you used, how you took the history to help generate your differentials?
   - Ask questions to help clarify why certain parts of the history were presented, why in that order.
3. Based on this history overall, what is your differential diagnosis and why?
   - If not covered by the candidate ask:
     - Please prioritise your differential in order of likelihood.
     - Which is most likely? – What in the history suggests this?
     - What red flag features did you consider?
     - What in your history has supported each differential?
     - Is there information that does not fit with your main diagnosis or alternatives?
     - What diagnoses have you excluded and why?
4. What examinations would you now conduct and why?
5. If student has not identified in their differential x/y/z, say to them: In addition to your differential, other possibilities include…. You then examine the patient which reveals____
   - How does this affect your differential list?
6. What tests should be requested? What would you be looking for?
   - Student should prioritise the tests in line with clinical practice/ guidance.
