# Supplementary material for: Assessing clinical reasoning in the OSCE: pilot-testing a novel oral debrief exercise
Source: BMC Med Educ. 2023 Oct 3;23:718. doi: 10.1186/s12909-023-04668-5 (PMC10548592; doi:10.1186/s12909-023-04668-5)
Supplement: Supplementary file 3 — Additional file 3. Individual interview semi-structured topic guide [file 12909_2023_4668_MOESM3_ESM.docx]

**Supplemental File 3. Individual interview semi-structured topic guide**

First complete the ‘Think Aloud’ phase of the interview for all 3 videos. Then explore:

- We will look at each marking domain in detail, but what are your overall impressions of the marking domains as a way of assessing the components of clinical reasoning?
- Looking at the first domain, “Identify & Summarize the patient’s problem”?
  - How did you find scoring this domain for each student?
  - Did all the elements make sense?
  - if not, why not ?
  - Is there anything missing in the domain elements?
- Looking at the second domain, “Prioritize a diagnosis (discriminate), can't miss diagnosis and alternatives”?
  - How did you find scoring this domain for each student?
  - Did all the elements make sense?
  - if not, why not ?
  - Is there anything missing in the domain elements?
- Looking at the third domain, “Management Planning”?
  - How did you find scoring this domain for each student?
  - Did all the elements make sense?
  - if not, why not ?
  - Is there anything missing in the domain elements?
- How useful were the marking domains in informing your global judgement?
- How did you feel scoring this oral debrief without having seen the consultation?
  - Do you think this would have helped or not?
- Overall, how well do you think this OD proposal assesses the student’s clinical reasoning?
  - Why / why not?
- How would you see this oral debrief fitting in to the medical education program for undergraduate students?
